# Incipient Mental Disease

**Published:** 1920-09-18

**Authors:** 


The Hospital

The Workers' Newspaper of Administrative Medicine and Institutional Life, Administration, National Insurance and Health.

No. 1789, Vol. LXVIII. SATURDAY, SEPTEMBER 18, 1920. PRICE TWOPENCE

### INCIPIENT MENTAL DISEASE.

It has been realised for a considerable time that some alteration should be made in regard to the early treatment of mental disorders; and already a certain number of psychiatric clinics have been instituted in order that patients may have the benefit of skilled advice with a view of obviating, if possible, the more acute symptoms of the disease, and, therefore, rendering it unnecessary to resort to certification and deprivation of liberty. Such out-patient departments and clinics are, however, of only limited utility. It is in-patient treatment that is so essential, even in many of the milder cases. The financial aspect is an important one: and it is difficult for the poor, or even for persons of limited means, to obtain the necessary treatment. In due course some satisfactory arrangement will no doubt be made. In the meantime there arises the question of treatment of these early cases in nursing homes and other places without the resort to certification. It is well known that such cases are already treated in large numbers under these conditions; and the suggestion, therefore, is not really a revolutionary one, but is rather a legalisation of what has had to be carried on to a great extent *sub rosa*.

The intentions of the framers of the new Bill* are no doubt excellent in regard to "treatment for incipient mental disorder." It is, however, a little difficult to speak definitely on the matter because part of it is set out in such ambiguous phraseology. Where it is clear what the meaning is, it is difficult to commend all the suggested changes in legislation. Take, for example, this clause: "Notwithstanding the provisions of any Act a person shall not, if the required conditions are complied with, be liable to any penalty for receiving to board, lodging, or taking charge of for a period not exceeding six months, or such longer period not exceeding in all twelve months as may be approved by the Minister, and whether for payment or not, any person suffering from mental disorder which is incipient in character and of recent origin, but not being a person who has been certified as a lunatic under the Lunacy Acts 1890 to 1911, or in respect of whom an order has been made under the Mental Deficiency Act 1913." Up to the words "of recent origin" this is very much to the point: but what are we to infer from the last part of the sentence? Does it mean that any person who has ever been certified is excepted from the scope of the Bill? If someone had a mental breakdown twenty years ago and required then to be placed under certificates, and if he—or she—now has a recurrence of the trouble, perhaps of less acuteness, can advantage not be taken of the proposed legislation? And if it means a person who is actually certified at the moment, how does it square with the provisions of the Lunacy Act of 1890, which expressly allow of certified patients being kept in private care?

In the event of the patient wishing to leave the home he has to give notice in writing, either to the person in charge or to the Minister, that he desires to be discharged. This is quite a wise provision: but here, as elsewhere, account is not taken of those who are unable to decide the matter for themselves: for example, the numerous cases of mental confusion—a type very common among soldiers during the recent war—in which there is temporary clouding of consciousness, perhaps for only a few weeks. On the other hand, it may be said that such cases would not have the chance of being nursed under these conditions. It is at present possible for a person to be admitted into a licensed house on his own application supported by the certificate of a medical man stating that the patient is not certifiably of unsound mind. Permission has to be obtained from the Board of Control to whom these papers have to be sent. That is to say, a person cannot enter directly into such an establishment unless a certain amount of latitude is allowed and he is taken in as a guest pending the consent of the Board arriving. The new Bill says that "no person shall be received into the institution, home or house, except with his previous consent in writing, and except on a certificate in writing by two duly qualified medical practitioners to the effect that that person is reasonably likely to benefit by treatment therein." When, in addition to this, we find that the reception of any person has to be reported to the Minister it is possible to realise that in future patients suffering from incipient mental disorder will practically be placed in the position of certified patients at the

* Ministry of Health (Miscellaneous Provisions) Bill. (London: H.M. Stationery Office.)

present time. True, there is no magistrate's order: but that is now a mere formality that could easily be dispensed with.

Enough has been said to make it clear that the Bill is by no means ideally conceived. It may be that over-scrupulous care to guard against the liberty of the subject being infringed, and the lingering suspicion against those who have charge of the mentally disordered, accounts for these stringent precautions. But in the first place the houses into which patients may be received have to be approved by the Minister, and secondly, they are to be periodically inspected by "officers appointed for that purpose" by him. These are excellent precautions and should be resented by nobody—if the visitations are carried out tactfully. The visitors, if they know their work, should soon be able to decide which places are satisfactory.

As to these officials—is there to be another visiting body in addition to the Board of Control? No mention is made of the Board. Are we to go through the same ridiculous phase as we did during the war, going on the assumption that these cases are not to be in any way associated with those who are tainted with a knowledge of insanity! As a matter of fact many—if not most—of the homes where such patients are taken are already known to and visited by members of the Board, for one or two certified patients are under care in some of these houses, and consequently they are subject to visitation. Why not, in view of the increase of work entailed, add to the membership of the Board of Control, or appoint inspectors as under the Mental Deficiency Act?

These criticisms are not made in a carping spirit. Indeed, it is pleasant to see that at last a move is being made in this matter.

